# The Human Dive Reflex During Consecutive Apnoeas in Dry and Immersive Environments: Magnitude and Synchronicity

**DOI:** 10.3389/fphys.2021.725361

**Published:** 2022-01-04

**Authors:** Michael Nordine, Anton Schwarz, Renana Bruckstein, Hanns-Christian Gunga, Oliver Opatz

**Affiliations:** ^1^Charité Universitätsmedizin Berlin, Corporate Member of Freie Universität Berlin, Humboldt-Universität zu Berlin, and Berlin Institute of Health, Clinic for Anesthesiology, Campus Benjamin Franklin Berlin, Berlin, Germany; ^2^Monash School of Medicine, Monash University, Clayton, VIC, Australia; ^3^Charité–Universitätsmedizin Berlin, Corporate Member of Freie Universität Berlin, Humboldt-Universität zu Berlin, and Berlin Institute of Health, Institute of Physiology, Center for Space Medicine and Extreme Environments Berlin, Berlin, Germany

**Keywords:** human dive reflex, consecutive apneas, cardiovascular regulation, cardiovascular synchronisation, human physiology

## Abstract

**Introduction:** The human dive reflex (HDR), an O_2_ conserving reflex, is characterised by an interplay of central parasympathetic and peripheral sympathetic reactions, which are presumed to operate independently of each other. The HDR is fully activated during apnoea with facial immersion in water and complete immersion in water is thought to increase the magnitude of HDR during consecutive apnoeas. A comparison of HDR activity between consecutive apnoeas in full-body immersion with consecutive apnoeas in dry conditions has not been fully explored. Also, the interplay between parasympathetic and sympathetic reactions involved in the HDR has not been thoroughly analysed.

**Methods:** 11 human volunteers performed 3 consecutive 60 s apnoeas with facial immersion in dry conditions (FIDC) and 3 consecutive apnoeas with facial immersion in full immersion (FIFI). Heart rate (HR), R-R interval (RRI), finger pulse amplitude (FPA), splenic width (SW) and SpO_2_ were all measured before, during and after apnoeas. A one-way ANOVA using Dunn’s *post hoc* test was performed to assess HDR activity, and a Pearson’s correlation test was performed to assess HDR synchronisation between physiological parameters during both conditions.

**Results:** Although HDR activity was not significantly different between both conditions, HR and RRI showed progressively greater changes during FIFI compared with FIDC, while SW and FPA changes were relatively equivalent. During FIDC, significant correlations were found between SW & SpO_2_ and FPA & SpO_2_. During FIFI, significant correlations were found between RRI & FPA, SW & FPA, HR & SpO_2_ and FPA & SpO_2_.

**Discussion:** While there was no significant difference found between HDR activity during FIDC and FIFI, consecutive apnoeas during FIFI triggered a greater magnitude of cardiac activity. Furthermore, significant correlations between RRI and SW with FPA indicate a crosstalk between parasympathetic tone with splenic contraction and increased peripheral sympathetic outflow during FIFI compared to FIDC. In conclusion, HDR activity during consecutive apnoeas does not differ between FIDC and FIFI. There appears to be however a greater level of synchronicity during apnoeas in FIFI compared to FIDC and that this is most likely due to the physiological effects of immersion, which could induce neural recruitment and increased cross talk of HDR pathways.

## Introduction

The human dive reflex (HDR), is an innate defensive reflex, which preserves O_2_ supply to critical organ systems, such as the brain and heart, during periods of apnoea (Foster and Sheel, 2005). The HDR is composed of 3 basic reflex arcs: sympathetic vasoconstriction, parasympathetic bradycardia and splenic contraction (Panneton and Gan, 2020). All these mechanisms ensure sufficient oxygenation to the brain during apnoea *via* the centralisation of blood volume, maintaining cardiac output and increasing O_2_ carrying capacity through increased serum haematocrit concentration (Alboni et al., 2011). The classic response of bradycardia also acts as an O_2_ conserving mechanism for the heart, particularly the highly O_2_ sensitive myocardial tissue (Marabotti et al., 2013), which ensures continued cardiac function while diving, while minimising O_2_ consumption in the periphery, such as muscle tissue.

The HDR is primarily triggered upon sub-maximal inspiration and subsequent facial immersion in water which activate distinct neural network pathways (Panneton, 2013). Upon initiation of apnoea, increased peripheral vasomotor activity, as well as cardio-inhibitory reflexes are initiated in the nucleus tractus solitarius (NTS; Gooden, 1994). Facial immersion in water triggers an increase in vagal activity (Hayashi et al., 1997), which is also controlled from the NTS. The cardio-inhibitory signals received by the NTS are then transmitted to the rostral ventrolateral nucleus (RVL), while activation signals are then transmitted to the nucleus ambiguus, which then the efferent signals are relayed to their respective target systems (Fitz-Clarke, 2018). The simultaneous inhibition/activation of physiological responses, which must be orchestrated properly for the HDR to be effective during periods of apnoea.

Upon activation of the HDR axis, heart rate (HR) decreases, thereby increasing R-R interval (RRI), which are primarily vagally controlled (Foster and Sheel, 2005). Peripherally, increases in peripheral vascular resistance (PVR) in selected arterioles occur (Foster and Sheel, 2005). Splenic contraction also occurs, known also as the haematological sequence of the HDR, which is triggered *via* alpha-adrenergic pathways which stimulate the sympathetic fibres surrounding the splenic capsule (Stewart and McKenzie, 2002). Splenic contraction is theorised to increase haematocrit by 4% in times of physiological stress, which would increase O_2_ supply (Stewart and McKenzie, 2002).

Given the common initial neural pathways involved in the HDR (NTS), these aforementioned factors should synchronise, to fully maximise the reflex. Given the inactivation/activation of sympathetic/parasympathetic pathways, there should occur a significant correlation between cardiac, vascular and haematological factors involved in the HDR. Previous studies examining the HDR, appear not to have examined the correlation/synchronisation of these multiple factors during dry or immersive apnoeas (Baković et al., 2003; Schagatay et al., 2004; Baranova et al., 2017; Elia et al., 2020).

The magnitude of the HDR is also dependent upon environmental influences, namely facial immersion in dry or submerged conditions, as well as ambient air and water temperature (Bosco et al., 2018). Complete immersion in water, leads to a centralisation of blood volume due to hydrostatic pressure, even before HDR has been activated (Schipke, 2001; Bosco et al., 2018). Triggering the HDR in immersive environments has the potential to maximise the physiological effect, compared with HDR in dry environments, due to potential neural recruitment, and the physiological priming effects of hydrostatic forces. This is a logical evolutionary advantage, as HDR in immersive environments would allow for deeper and longer dives.

Serial apnoeas lead to consecutive triggering of the HDR, and presumably, the HDR would become progressively stronger with each apnoea cycle. However, previous studies have found that cardiovascular HDR responses do not become progressively stronger during serial apnoeas, whereas splenic contraction does become progressively stronger (Schagatay et al., 2004). To our knowledge, no study has compared cardiovascular and splenic changes during consecutive apnoeas during facial immersion in dry and full-body immersive environments to assess if the magnitude of HDR progressively increases.

Given that the HDR is influenced by environmental conditions, such as the HDR being significantly stronger during apnoea with facial immersion compared with apnoea in dry environments (Hurwitz and Furedy, 1986), we hypothesised that the physiological factors of HDR would be fully activated in full-body immersion apnoeas compared to facial immersion apnoeas in dry environments. Also, these factors should become progressively stronger during consecutive apnoeas in full-body immersion. We further hypothesised that the factors involved in the HDR would show a higher level of synchronisation during immersive apnoeas compared with dry apnoeas, due to the physiological effects of hydrostatic forces augmenting the HDR. The overarching goal of this study was to determine if the magnitude of the factors involved in the HDR during serial apnoeas would differ between dry and immersive environments, as well as determining if the physiological parameters involved in the HDR are synchronised or operate independently during serial apnoeas in both conditions, among non-apnoea trained participants. The results will provide more insights concerning environmental influence and physiological cross talk for future HDR physiological research.

## Materials and Methods

The study was conducted at the Institute of Physiology, Center for Space Medicine and Extreme Environments, Charité, Universitätsmedizin, Berlin, Germany in the summer of 2015 and was approved by the Charitè ethics committee (EA2/024/15). For this study, 11 participants were recruited. No participants were experienced divers, nor had any participant undergone any apnoea training prior to this study. Participants were asked to provide an estimation of their daily activity level on a scale of 1–10 upon recruitment (1–3 sedentary, 4–5 slightly active, 6–7 moderately active, 8–9 active and 10 very active).

The study was divided into 2 parts: facial immersion in dry conditions (FIDC) and facial immersion in full immersion (FIFI). Both parts were performed in the immersion study room, which contained an 2 × 1 × 1 m immersion tank filled with thermoneutral water. Each participant wore a neoprene short sleeve 3 mm dive-suit during both parts. The arms and legs were exposed for each participant. Also, a square-sized hole was made in the wetsuit, which left the entire left flank exposed on order to minimise interference during splenic measurements. Cardiovascular monitoring equipment consisted of a chest strap with a mobile heart rate monitor capable of measuring RRI (Polar S810, Polar Electro Oy, Kempele, Finland), pulse oximeter worn on the left earlobe and a pulse amplitude analyser worn on the left index finger using piezoelectric material. The Piezoelectric sensor is a polyvinyl difluoride-based system, which reconstructs a pulse wave form. The signal is constructed from converted pressure signals measured from the left index finger arterioles, into a change of strain-dependent resistance which produces a voltage signal in millivolts, observed as a pulse wave (i.e. the dynamic mechanical pressure derived from the pulse wave exerts changes in electrical conductance in the sensor; Wang et al., 2020). This signal was used as a surrogate PVR marker, which was not calibrated prior to testing and will be referred to as finger pulse amplitude (FPA).

HR and RRI from the Polar unit are reported as beats/min and milliseconds. FPA is reported as mV. Splenic measurements were recorded using a GE portable sonography device (GE Logiq e Ultrasound, GE Medical Systems Inc.) and are reported as centimetres. HR, RRI and PVR were recorded *via* Bluetooth using the Heally System (Spacebit GmBH, Wiesbaden, Germany) with Healthlab explorer (Spacebit GmBH, Wiesbaden, Germany) being used to analyse and record data.

For both parts of the study protocol, FIDC and FIFI, 3 consecutive apnoeas were undertaken, in which the participant was requested to hold their breath for as long as they could, for maximal 60 s. Prior to each apnoea, the participant exhaled completely, inhaled to sub-maximal inspiration and then immersed their head in water. A bucket of thermoneutral water was used for FIDC apnoea, while each subject immersed their face in the thermoneutral water in the immersion tank for FIFI. A nose clip was used during the entire study. Prior to apnoeas, a 5-min baseline measurement was performed, as well as a 5-min post apnoea measurement. Between FIDC and FIFI, each participant rested for 2 h. Water temperature in both conditions was kept at thermoneutral temperatures (35°C).

During the first part of the study (FIDC), the participant sat upright, and for each apnoea, the participant lowered his/her head into a water filled basin. Between the 3 consecutive apnoeas, participants performed only one exhalation and one inhalation as preparation for the next apnoea. For the second part of the study (FIFI), each participant immersed themselves up to the neck in water, while sitting in a water-proof chair.

Cardiovascular parameters were monitored continuously during the study. Splenic measurements were performed every 60 s during the pre/post apnoea phase and every 10 s during apnoea. To perform splenic measurements in such short consecutive time slots, our study used measurement of splenic width (SW) as an indicator of splenic activity. The finger and ear sensors were not immersed during both parts and for all apnoeas. The finger of the subject was either resting on a table during FIDC and resting on the side of the immersion tank during FIFI. The sonographic probe was covered in a plastic cover during both measurements, while sonographic gel was used for FIDC measurements. Splenic measurements during FIFI did not require the use of sonographic gel. The researcher performing the splenic measurements was not in the immersion tank with the participant.

For statistical analysis, the median and range for all phases are reported. During apnoea, data from the last 5 s of apnoea were used to reflect maximal HDR effect.

For multiple phase comparisons, a one-way ANOVA was performed, with an emphasis on comparing all values from baseline. Due to the small sample size (*n* = 11) and non-parametric data, a Kruskal-Wallis test was performed with Dunn’s *post hoc* test. To test for synchronicity of HDR factors, a correlation matrix was created for all parameters during dry and immersive conditions, using Pearson’s correlation test. Statistics were performed using JASP software (JASP Team, Version 0.14.1), with a significance set to *p* < 0.05. Values are reported as median with min-max ranges due to the small sample size and non-normality of data. For correlation analysis, the R^2^ value is reported. Graphics were created with Data Graph software (Visual Data Tools, Inc., Version 4.6).

## Results

All 11 participants (6 female, 5 male) completed the study protocol without interruption. Demographic information and activity level is presented in Table 1. Aside from height, no significant differences were found between females and males. Females reported a median higher daily activity level than the males of the cohort, however, this was not statistically different.

**Table 1:** Cohort demographic variables.

Baseline factor	All	Female/Male
Age	24 (21–42)	22 (21–26)/27 (23–42)
Height	178 (163–186)	173 (163–179)/180 (175–186)^*^
Weight	67 (55–92)	61 (55–70)/76 (58–92)
BMI	21.5 (18.9–26.6)	21.3 (19–24.2)/23.3 (19–26.6)
BSA	1.81 (1.58–2.18)	1.74 (1.58–1.85)/1.95 (1.68–2.18)
Activity level	6 (2–10)	7 (4–10)/4 (2–7)

* *Significantly greater*.

**Figure 1:**
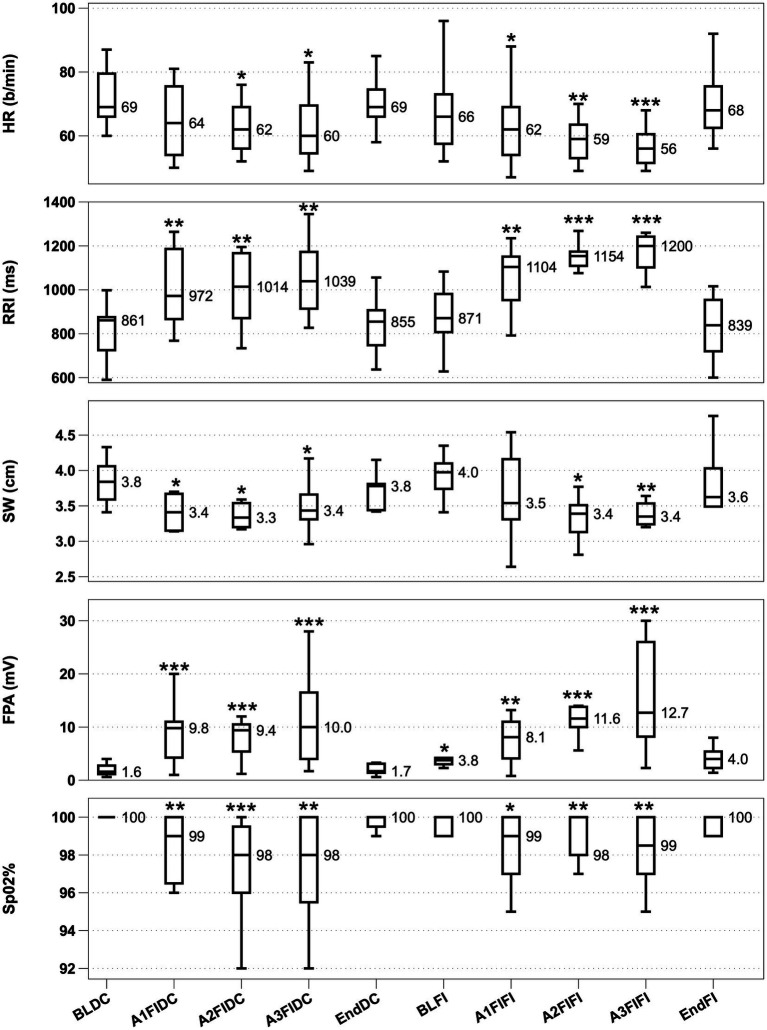
HDR activity from top to bottom with significance denoted. From top to bottom, Heart rate (HR; **A)**, R-R Interval (RRI; **B)**, Splenic width (SW; **C)**, Finger Pulse Amplitude (FPA; **D)** and O_2_ saturation (SpO_2_; **E)**. Boxplots display interquartile range with a median value reported. ^*^p < 0.05, ^**^p < 0.01 and ^***^p < 0.001. BLDC (Baseline dry condition), A1-A3 FIDC (Apnoeas 1–3 facial immersion dry condition), EndDC (end of apnoea sequence dry condition), BLFI (Baseline full immersion), A1-A3 FIFI (Apnoeas 1–3 full immersion, facial immersion) and EndFI (End of Full immersion).

Mean breath-hold time (BHT) for the 3 consecutive apnoeas during FIDC was 45 s ± 5.1, 47 s ± 4.3 and 51 ± 3.4. Median BHT for the 3 consecutive apnoeas during FIFI was 52 s ±4.0, 53 s ±3.3, 54 s ±3.3. BHT between FIDC and FIFI revealed no differences. Figure 1 shows HDR parameter phase-based activity during the study as box plots.

HR showed significant decreases from baseline in 2 out of 3 apnoeas during FIDC and during all 3 apnoeas throughout FIFI, with the HR progressively decreasing during FIFI apnoeas. RRI showed significant increases in all 3 apnoeas during both conditions, with RRI during FIFI becoming progressively longer. SW significantly decreased in all 3 apnoeas in FIDC and 2 out of 3 apnoeas in FIFI. FPA amplitude increased significantly during all apnoeas, while also being significantly elevated during immersion prior to apnoea. SpO_2_ was significantly decreased in all apnoeas during both conditions. Water immersion did not appear to interfere with signal quality from the HR/RRI monitor. The finger and ear sensors were kept dry during the entire study.

Table 2 displays pairwise correlations between HDR parameters for FIDC and FIFI. The only significant correlations found during FIDC were SW/SpO_2_ and FPA/SpO_2_. During FIFI, significant correlations were found between HR/SpO_2_, RRI/FPA, RRI/SpO_2_, SW/FPA and FPA/SpO_2_.

**Table 2:** Pairwise correlations (*R* and *p* values) between HDR parameters during FIDC (right) and FIFI (left).

HDR pair	Pearson’s *R* FIDC	*p* value FIDC	Pearson’s *R* FIFI	*p* value FIFI
HR/SW	0.05	0.72	−0.06	0.68
HR/FPA	0.05	0.7	−0.01	0.94
HR/SpO_2_	−0.04	0.75	0.3	0.03^*^
RRI/SW	−0.04	0.81	−0.13	0.39
RRI/FPA	0.08	0.57	0.33	0.02^*^
RRI/SpO_2_	−0.02	0.86	−0.48	<0.001^***^
SW/FPA	−0.03	0.84	−0.39	<0.01^**^
SW/SpO_2_	0.4	<0.01^**^	−0.01	0.95
FPA/SpO_2_	−0.48	<0.001^***^	−0.33	0.02^*^

*Significance is denoted as*

* *p < 0.05*;

** *p < 0.01; and*

*** *p < 0.001*.

## Discussion

This study demonstrated that during consecutive apnoeas, the measured physiological parameters involved in HDR exhibited equivalent activity during consecutive apnoeas in FIDC and FIFI. Although no significant differences were found between the two conditions, consecutive apnoeas during FIFI triggered a progressive decrease in HR during all 3 apnoeas with a progressively increasing RRI. FPA was significantly increased equally during both conditions. During FIDC, SW significantly decreased in all 3 apnoeas, whereas this was only present in the last 2 apnoeas during FIFI. Therefore, the components involved in the HDR were activated relatively equally during consecutive apnoeas in both conditions, however, the magnitude of the parasympathetic response seems to be a greater during immersive rather than dry conditions. The synchronisation of the HDR factors exhibited unique significant correlations depending on the environmental condition, with 5 pairwise correlations being observed in FIFI compared to 2 during FIDC. During FIDC, SpO_2_ was correlated with SW and FPA, suggesting that the vascular and haematological components of the HDR are at least responding in some part to chemoreflexive stimuli (falling SpO_2_). During FIFI, correlations were found between RRI and SW with FPA, which suggest that immersive apnoeas can trigger a synchronisation of parasympathetic tone with peripherally mediated sympathetic tone, which were not present in FIDC. Furthermore, the chemoreflexive correlation between SW was not present in FIFI, rather, SpO_2_ was correlated with HR and FPA. The differences in HDR synchronisation are most likely due to the hydrostatic effects of water immersion, which could play a role in heightened neural recruitment.

## Mechanisms of Hdr: Non-Immersive Vs. Immersive Conditions

Significant changes in HR, RRI, FPA and SW were observed in both conditions, with a tendency for the parasympathetic mechanisms (HR and RRI) during FIFI to show a greater magnitude than in FIDC. This is best explained by the environmental conditions, and not by the actual apnoeas, as the apnoeas were identical in technique and total time during both conditions. Immersion in water triggers an increased intrathoracic volume, and central venous pressure *via* centralised blood shift, leading to an increased cardiac output and stroke volume (Risch et al., 1978). The displacement of this blood volume increases cardiac volume by about 180 ml, which contributes to an increase in atrial volume (Lange et al., 1974). RRI increases in water immersion due to this centralisation of volume (Schipke, 2001), while the hydrostatic pressure leads to venoconstriction, leading to increases in mean arterial pressure and pulse pressure (Pendergast et al., 2015). This physiological change is the equivalent of an autotransfusion, which may negate the need for splenic contraction initially (Pendergast et al., 2015). Due to the physiological effects of water immersion, (centralisation of blood volume), splenic contraction would not necessarily need to occur in the first FIFI apnoea, rather splenic contraction occurs during subsequent apnoeas in water. Other working groups have observed significant splenic contraction during repeated apnoeas (Baković et al., 2003) and that splenic emptying occurs because of repeated apnoeas (Elia et al., 2020). Serial apnoeas in facial immersion alone can elicit an increase in haematocrit, which is attributed to splenic emptying (Stewart and McKenzie, 2002). The observation of no noticeable differences in SW reduction between FIDC and FIFI would suggest that splenic contraction is not necessarily dependent on full water immersion per say, rather due to the triggering of HDR *via* facial immersion alone. Also, splenic contraction in humans during apnoea is triggered by alpha-adrenergic catecholamine stimulation, rather than splenic nerve stimulation (Stewart and McKenzie, 2002). During repeated apnoeas, splenic volume among non-trained breath-hold divers progressively decreased, while all other HDR cardiovascular factors did not show progressive changes, suggesting that these progressive splenic volume decrease enable longer breath-holding for subsequent apnoeas (Baković et al., 2003). Elia et al. recently published their findings showing that during consecutive apnoeas in total body immersion, non-trained breath-hold divers exhibited non-significant decreases in HR, whereas splenic volume did significantly decrease in 3 out of 5 apnoeas. They concluded that the reduction in splenic volume is due to hypoxic stress, and therefore, the spleen is triggered by chemoreflexive, rather than baroreflexive mechanisms (Elia et al., 2020). The resulting splenic contraction after serial apnoeas does appear to significantly increase plasma erythrocyte count in trained and non-trained apnoea, although this increases in much more pronounced in trained apnoea divers (Baković et al., 2005). Our study did not measure directly erythrocyte counts, so no conclusion can be made if in fact this splenic contraction seen in our study impacted haematological values.

Results from earlier studies observing the differences in cardiovascular mechanisms during prolonged apnoeas in dry vs. immersive environments have found that end-systolic volume is greater in immersion apnoea than dry apnoea (Marabotti et al., 2013), while no differences in HR changes were found. Decreases in HR and increases in arterial pressure have also been found to be no different during apnoeas in dry vs. immersive environments, if apnoea is performed with full facial immersion in water (de Bruijn et al., 2009). Our findings can confirm these findings, as facial immersion alone appears to be an adequate HDR trigger; however, the environmental condition can affect the magnitude of the HDR response regardless of the environmental condition (de Bruijn et al., 2009).

The main reasons as to why HR and RRI exhibited a greater response magnitude during FIFI is most likely due to the physiological effects of water immersion (fluid shifts), as water immersion stimulates an increase in RRI, which is primarily parasympathetically modulated (Schipke, 2001), although in our cohort, immersion alone did not seem to effect RRI. Apnoeas in FIFI have been observed to trigger higher vagal tone, leading to greater HR decreases (Foster and Sheel, 2005). In a previous study, during 3 repeated maximal apnoeas, HR, MAP and skin blood flow, although significantly different from baseline, did not progressively change, whereas splenic volume did appear to progressively decrease, along with increasing haematocrit (Schagatay et al., 2004). Therefore, based on these previous findings, it is thought that splenic emptying acts as a promotor for increasing BHT and O_2_ conserving ability, while the cardiovascular mechanisms of the HDR do not differ during serial apnoeas.

In this study, FPA increased significantly throughout apnoeas in both conditions equally. Peripherally mediated vasoconstrictive reflex of the HDR only requires facial immersion and/or apnoea for activation; however, FPA was also significantly increased upon immersion, which would suggest that immersion alone can activate the peripheral mechanism of the HDR prior to apnoea onset. Heightened arteriolar resistance has been recorded during breath holds while completely immersed compared to dry condition apnoeas (Craig, 1963). Water immersion can trigger baroreflex stimulation, which would explain the initial significant increase in FPA during immersion baseline, and the subsequent FPA increases during FIFI (Lindholm and Lundgren, 2009). Thermoneutral water induces significant venodilation in the lower extremities, which can further induce a refractory arteriole constriction, which would explain the increased FPA during immersion (Beliard et al., 2017). Immersion causes a shift of 500 ml of blood flow to the intrathoracic circulation, which would induce an increase in vascular activity, as well as affecting the degree of vagal tone and vasomotor activity during apnoeas in immersive environments (Bosco et al., 2018). Finally, vascular activity during immersion is highly selective, with increased vasomotor activity in the periphery and vasodilation in the visceral organs, which may account for a transient decrease in overall systemic vascular resistance (Krasney, 2011).

Costalat et al. found that the O_2_ conserving breaking point for non-trained breath-holding divers occurs halfway through apnoea time, which is at the time point where HDR activity rapidly increases (Costalat et al., 2016). In our cohort, BHT was not different between FIDC and FIFI, suggesting that full immersion does not play a role in enabling longer breath-holding, and rather, individual factors involved in the HDR activity play a role in influencing BHT to 60 s, as well as participants presumably becoming more accustomed to subsequent breath holds. Total immersion apnoea in elite divers triggered a maximal HR decrease and RRI increase at 60 s breath hold, which plateaued afterwards, meaning that 60 s appears to be adequate to trigger full HDR cardiac activity (Kiviniemi et al., 2012). Had our cohort performed apnoeas for longer than 60 s, a possible higher magnitude of HDR activity may have been observed; however, this is speculation. On the other hand, HR responses during HDR activation plateau from 30 s from start of apnoea (Yamaguchi et al., 1993).

The HDR is also highly individual-dependent, with non-uniform responses among humans, which suggests genetic polymorphism. In a study with 80 participants, consecutive FIDC apnoeas did not significantly decrease HR, but triggered uniform increases in PVR. The intensity of this vascular response was found to be highly dependent upon genotype influencing the renin-angiotensin and kinin systems (Baranova et al., 2017). Other findings have indicated that the HR decreases during apnoea are ubiquitous among all humans and are not individually dependent, which would counter the findings of Baranova et al. (Ferretti and Costa, 2003). This study could show that the measured vascular component (FPA) was consistently active during all apnoeas regardless of conditions, whereas HR activity showed progressive declines during FIFI, thereby supporting the findings of Baranova et al.

Finally, although breath-hold time between FIDC and FIFI was not statistically different, there was a tendency for BHT to progressively increase for subsequent apnoeas. This trend has also been recorded previously and is explained in partly due to participants getting accustomed to longer breath holds, while O_2_ debt seems to increase during each subsequent apnoea (Heath and Irwin, 1968). The slightly longer, albeit, non-significant differences between BHT during FIFI compared to FIDC is possibly due to immersion causing a delay in CO_2_ build-up (Sterba and Lundgren, 1985).

## Synchronisation of the Hdr: Fidc Vs. Fifi

The results of this study found that significant correlations exist between the vascular pathway of the HDR (FPA) with vagal tone (RRI) and splenic contraction during FIFI compared with FIDC. This suggests that apnoeas in immersive environments induce a cross talk between these systems, allowing for a higher level of synchronisation during FIFI than FIDC. While FIDC apnoeas did trigger a significant change in FPA, HR, RRI, SW and SpO_2_ these factors appear to operate independently from each other. The only significant correlations were between SpO_2_/FPA and SpO_2_/SW. This suggests that during apnoeas in dry environments, the vascular mechanisms are stimulated *via* chemoreflexive pathways due to falling SpO_2_. This has also been confirmed by prior breath-hold studies, which found that the increases in vascular resistance and bradycardia are most likely mediated by chemoreflexive pathways *via* breath-holding (Brick, 1966). Also, previous studies have shown that mild hypoxia can elicit splenic emptying (Stewart and McKenzie, 2002). The splenic expulsion of stored red blood cells can lead to a 10% increase in arterial O_2_ content (Stewart and McKenzie, 2002), which could explain also the positive significant correlation between SW and SpO_2_ during FIDC.

During FIFI, significant correlations were found between HR/SpO_2_, RRI/FPA, RRI/SpO_2_, SW/FPA and FPA/SpO_2_. Apnoea in immersive environments seems to allow for a greater neural network synchronisation of the sympathetically mediated vasoconstriction and the parasympathetically mediated vagal tone. While no correlation was found between HR and PVR, FPA and RRI did show a moderate significant correlation, which would indicate that the interplay between central parasympathetic and peripheral sympathetic drive coordinate during underwater diving. The moderate significant negative correlation between FPA and SW does suggest that splenic activity is linked to sympathetic vasoactive inputs, rather than vagal tone as evidenced by previous research (Espersen et al., 2002).

The greater synchronisation of HDR factors during FIFI can also be explained by a greater central integration of the HDR. This central integration relay station, situated in the dorsal medullary horn of the trigeminal nerve, receives the peripheral afferent signals of apnoea/environmental cues and the following efferent output activates the HDR to a greater extent than in dry conditions (Panneton, 2013). This could also explain the greater synchronisation of HDR factors during FIFI. Water immersion, compared to dry environments, leads to an increase in plasma volume *via* the translocation of intracellular to intravascular volume to the thoracic cavity (Gauer and Henry, 1976; Pendergast et al., 2015). Afferent signals from the periphery are received at the nucleus solitarius which is embedded in the medulla oblongata, and consequently, 2 distinct pathways are activated. One pathway is mediated by the nucleus ambiguus, which triggers the parasympathetic arm of the HDR (HR, RRI). The other arm flows to the rostral ventral lateral medulla complex, which efferently activates the peripheral sympathetic drive of the HDR (increase of PVR). Both pathways lead to an increase of mean arterial pressure (MAP). It is of interest that these two arms do not appear to intersect, beyond their origination point in the nucleus ambiguus (Choate et al., 2014). One would expect to see at least a moderate correlation between HR and FPA based on the common dual cholinergic pathways to the cardiac system and the adrenergic pathways to the vasomotor system, as these factors have been shown to independently increase BHT during apnoeas (Baranova et al., 2020). However, no correlation could be found between these 2 factors, although a correlation could be found between FPA and RRI during FIFI. These findings would suggest that despite cross talk with vagal tone and sympathetic HDR factors, heart rate seems to operate independently.

Finally, the link between the cardiovascular and haematological responses during apnoea is thought to not be linked, but consist of independent reflexes (Schagatay et al., 2007). This appears to be supported during FIDC, but not FIFI, where the significant correlation between SW and FPA exhibits some form of communication between these responses. Whether it is a purely independent operation or a neurally linked one remains to be investigated.

## Limitations

The primary limitation is that of a small sample size (*n* = 11). Also, our study included only non-apnoea trained participants, as apnoea trained individuals exhibit a specific HDR than non-apnoea trained divers, which includes a more pronounced bradycardia and increases in SVR activity (Tocco et al., 2012), as well as a increased splenic contraction (Prommer et al., 2007). Thus, the results of this study were from a homogenous cohort of non-apnoea trained divers, and the inclusion of apnoea trained divers may have impacted the results. Also, the specific goal of this study was to specifically examine non-apnoea trained individuals so that the results would apply to a broader segment of the population. A further limitation is that the apnoea protocol did not allow for maximal apnoeas and that apnoea time was held to maximal 60 s in order to maintain uniformity in the protocol. While the HDR could be induced in these 60-s intervals, longer apnoeas may have induced a stronger HDR responses. Another limitation of this study is the use of only SW to indicate splenic activity. This was done due to the narrow time window of maximal apnoeas, thereby negating the need to identify the caudal-cranial length of the spleen, which can be made difficult due to costal shadowing. Also, during immersion in apnoeas, the cranial border of the spleen was not visible in all subjects during the last 5 s of apnoea making complete splenic measurements difficult. Also, doppler views of the splenic vasculature would have been useful to solidify correlations between FPA and splenic contraction. Advanced cardiovascular monitoring may have led to more insights, as the availability of better methods could have enabled stroke volume and cardiac output monitoring. Finally, although cold water can induce a stronger HDR, specifically HR decreases (Gooden, 1994), thermoneutral water was used in the study as the immersion pool at our facility did not allow for water cooling. Furthermore, cold water contact with the trigeminal nerve can elicit the cardiovascular effects of the HDR (bradycardia, lengthening RRI and vascular resistance increases) even without apnoea (Johnson et al., 2017). The sole use of trigeminal stimulation *via* facial immersion in cold water without apnoea has been used for autonomic testing (Khurana and Wu, 2006) or as a countermeasure to orthostatic stress (Johnson et al., 2017). The intent of this study, however, was to utilise thermoneutral water to isolate the HDR solely based on apnoea and bypass the cold water HDR activation. Also, in order to isolate the mechanisms of the HDR triggered solely by facial immersion and apnoea would trigger splenic emptying and enable a correlation analysis. It remains to be explored if splenic emptying can be elicited solely by cold water trigeminal stimulation without apnoea. Also, had cold water been used either during FIDC or FIFI, the HDR effects would have been much more pronounced (Andersson et al., 2000). Finally, baseline physical activity was not uniform among the participants. Physical conditioning can affect autonomic functioning, as well as the HDR (Manley, 1990), which may have influenced our findings. A larger cohort, as well as grouping for physical activity, or explicitly examining a homogenous group of subjects with regards to physical activity may lead unique HDR activation and activity.

## Summary and Future Outlook

This study showed that the mechanisms of the HDR were stimulated nearly equally during consecutive apnoeas in both dry and immersive conditions. HR and RRI did seem to show a greater magnitude in immersive conditions, suggesting that the HDR is stronger during apnoeas in aquatic environments. The synchronicity of HDR factors were greater in number in immersive rather than dry conditions, and this is due to the physiological effects of water immersion, leading to increased neural recruitment and neural network cross talk among HDR pathways. The results from this small cohort do shed more light on the environmental influence and synchronicity of the HDR mechanisms in humans. Beyond that of purely physiological data, the consecutive apnoeas to elicit the HDR could be used prior to anaesthesia induction in order to promote splenic emptying and increase O_2_ availability, as well as increasing blood pressure during anaesthesia induction. Triggering the HDR could further prolong apnoea time in high-risk patient and could possibly maintain blood pressure during the induction period. Furthermore, clinical applications include autonomic testing to determine parasympathetic/sympathetic reactivity (Macartney et al., 2020) and potential HDR training prior to SCUBA or apnoea diving. In summary, the results of this study do shed light on the interplay of the HDR factors active in both conditions and warrant further investigations into these neural pathways supporting O_2_ conservation among humans in aquatic environments and beyond.

## Data Availability Statement

The raw data supporting the conclusions of this article will be made available by the authors, without undue reservation.

## Ethics Statement

The studies involving human participants were reviewed and approved by Charité-Ethics Committee. The patients/participants provided their written informed consent to participate in this study.

## Author Contributions

MN, AS, and OO performed the study. H-CG provided the research facility. MN and AS performed the statistical analysis. MN, AS, RB, and OO wrote and revised the manuscript. All authors contributed to the article and approved the submitted version.

## Conflict of Interest

The authors declare that the research was conducted in the absence of any commercial or financial relationships that could be construed as a potential conflict of interest.

## Publisher’s Note

All claims expressed in this article are solely those of the authors and do not necessarily represent those of their affiliated organizations, or those of the publisher, the editors and the reviewers. Any product that may be evaluated in this article, or claim that may be made by its manufacturer, is not guaranteed or endorsed by the publisher.
